# The Possible Impact of Vaccination for Seasonal Influenza on Emergence of Pandemic Influenza via Reassortment

**DOI:** 10.1371/journal.pone.0114637

**Published:** 2014-12-10

**Authors:** Xu-Sheng Zhang, Richard Pebody, Daniela De Angelis, Peter J. White, Andre Charlett, John W. McCauley

**Affiliations:** 1 Modelling and Economics Unit, Centre for Infectious Disease Surveillance and Control, Public Health England, London, United Kingdom; 2 Medical Research Council Centre for Outbreak Analysis and Modelling, Department of Infectious Disease Epidemiology, Imperial College School of Public Health, London, United Kingdom; 3 Respiratory Diseases Department, Centre for Infectious Disease Surveillance and Control, Public Health England, London, United Kingdom; 4 Statistics Unit, Centre for Infectious Disease Surveillance and Control, Public Health England, London, United Kingdom; 5 Medical Research Council Biostatistics Unit, University Forvie Site, Cambridge, United Kingdom; 6 NIHR Health Protection Research Unit in Modelling Methodology, Department of Infectious Disease Epidemiology, Imperial College School of Public Health, London, United Kingdom; 7 Medical Research Council National Institute for Medical Research, Mill Hill, London, United Kingdom; National Institutes of Health, United States of America

## Abstract

**Background:**

One pathway through which pandemic influenza strains might emerge is reassortment from coinfection of different influenza A viruses. Seasonal influenza vaccines are designed to target the circulating strains, which intuitively decreases the prevalence of coinfection and the chance of pandemic emergence due to reassortment. However, individual-based analyses on 2009 pandemic influenza show that the previous seasonal vaccination may increase the risk of pandemic A(H1N1) pdm09 infection. In view of pandemic influenza preparedness, it is essential to understand the overall effect of seasonal vaccination on pandemic emergence via reassortment.

**Methods and Findings:**

In a previous study we applied a population dynamics approach to investigate the effect of infection-induced cross-immunity on reducing such a pandemic risk. Here the model was extended by incorporating vaccination for seasonal influenza to assess its potential role on the pandemic emergence via reassortment and its effect in protecting humans if a pandemic does emerge. The vaccination is assumed to protect against the target strains but only partially against other strains. We find that a universal seasonal vaccine that provides full-spectrum cross-immunity substantially reduces the opportunity of pandemic emergence. However, our results show that such effectiveness depends on the strength of infection-induced cross-immunity against any novel reassortant strain. If it is weak, the vaccine that induces cross-immunity strongly against non-target resident strains but weakly against novel reassortant strains, can further depress the pandemic emergence; if it is very strong, the same kind of vaccine increases the probability of pandemic emergence.

**Conclusions:**

Two types of vaccines are available: inactivated and live attenuated, only live attenuated vaccines can induce heterosubtypic immunity. Current vaccines are effective in controlling circulating strains; they cannot always help restrain pandemic emergence because of the uncertainty of the oncoming reassortant strains, however. This urges the development of universal vaccines for prevention of pandemic influenza.

## Introduction

Two evolutionary events make influenza A viruses hard to control: antigenic drift due to mutation and antigenic shift generated from reassortment which occurs when two different influenza A viruses co-infect a host cell. Mutations, which cause relatively small but frequent changes in antigenicity of virus strains, are responsible for the seasonal influenza epidemics. Vaccines used to control seasonal flu must be reviewed twice each year in anticipation of the upcoming winter influenza season [1]. Because the vaccine only provides partial protection and was so far generally employed in an at-risk strategy, which therefore achieves only a minimal reduction in transmission in a population, influenza A viruses still cause much morbidity and mortality. Reassortment can lead to dramatic changes in the viral phenotype and is responsible for at least the three of the last four pandemics in humans in the 20^th^ and 21^st^ centuries [2], [3]. Reassortment events that produced pandemic strains in 1957 and 1968 likely occurred in people (e.g., [4], [5], [6]), but in 2009 reassortment was presumed to occur in pigs [7]; a recent study [8] infers that the 1918 pandemic H1N1 influenza A virus arose via reassortment between a pre-existing human H1 virus and an avian virus. Even though not as common as mutations, accumulated data suggest that it is much more frequent than was thought before [3], [9], [10], [11], [12]. From limited data sources we estimated that the average rate of reassortment is roughly 10^−5^ per coinfection per day [13].

In some instances, a reassortant virus can have high pathogenicity in animals and humans. The exchange of genes between pairs of influenza A virus subtypes increased virulence in animal models, including reassortment between subtypes H9N2 and H1N1, between H5N1 and H1N1, and between H3N2 and H5N1 [14], [15]. Reassortment events have historically introduced antigenically distinct subtypes for which there has been little previous infection and little cross-protection acquired from contemporary vaccine formulations. In 1957, reassortment between an avian H2N2 and the circulating H1N1 viruses precipitated an H2N2 pandemic [16]. The virus underwent further reassortment with an avian H3 virus to generate the H3N2 pandemic in 1968 [17]. The pandemic strains were more pathogenic than previously circulating seasonal influenza strains, and each of these pandemics is estimated to have killed in excess of one million people [4], [5]. A reassortant virus may also have high transmissibility within animal models [14], [15] and within humans. Whilst co-circulating with either seasonal H1N1 or H3N2 strains, the A(H1N1) pdm09 virus was able to outcompete these strains and become the dominant transmissible virus for two years [18]. Following the emergence of the A(H1N1) pdm09 virus, the circulating seasonal H1N1 strains were replaced in the human reservoir within a year. A similar phenomenon has been seen following the introduction of other pandemic virus strains into humans, whereby the new pandemic strain replaced a previously circulating subtype (reviewed by [19]).

Since pandemic influenza is caused by a novel strain, a delay of around 6 months is to be expected until vaccine starts to become available due to the time taken for the various manufacturing steps (e.g. [20], [21], [22]). Seasonal influenza vaccines may offer no direct protection, but they are used to reduce the prevalence of circulating strains and therefore that of their coinfection. As the generation of reassortant strains depends on coinfections of different influenza A viruses, it is natural to ask how a vaccination programme for seasonal influenza in humans might affect the chance of novel pandemic strain emergence via reassortment and the attack rate once it emerges.

Studies from animal models show that infection with influenza A viruses can induce partial heterosubtypic immunity and empirical analyses also indicate that immunity acquired from natural infection in humans can also partially protect the patients against other strains [21], [23], [24], [25], [26], [27], [28]. For example, Cowling et al. [26] have found that those infected with seasonal influenza A during the 2008–2009 season in Hong Kong had a lower risk of laboratory-confirmed A(H1N1) pdm09 infection. Our recent theoretical investigations show that cross-immunity induced by natural infection can greatly reduce the opportunity for pandemic emergence [13].

Two different types of seasonal influenza vaccines are available: inactivated and live attenuated. Both are safe and effective in inducing protective antibody responses against matching seasonal strains of influenza, but only live attenuated vaccines can induce heterosubtypic immunity [21], [29], [30], [31], [32]. For example, live attenuated vaccines in animals induce broad protective immune responses [33], [34] and their use in humans has also shown to induce CD4+, CD8+, and γδ T cells relevant for broadly protective heterosubtypic immunity [32]. Such vaccines have been used for some time in North America and have now recently been introduced into Europe, including in the UK as part of a new universal childhood influenza immunisation programme. Nevertheless, whether vaccination for seasonal influenza can provide any protection against pandemic strains is controversial. Katz et al. [35] show that vaccination with both types of seasonal influenza vaccines during 2005–2009 seasons was unlikely to provide protection against the A(H1N1) pdm09 infection. However, a systematic review by Yin et al. [28] suggests that trivalent influenza vaccines (TIVs) provided moderate cross-protection against laboratory-confirmed A(H1N1) pdm09 illness. Whereas, Skowronski et al. [36] found that previous vaccinations for seasonal influenza increase the risk of pandemic infection in 2009, which is unlikely to be explained by an unmeasured confounder [37]. Thus seasonal influenza vaccination may generate at most weak heterosubtypic immunity compared to that induced by natural infection [38]. In view of this, effective vaccination of children against seasonal influenza A viruses might prevent the induction of heterosubtypic immunity by natural infection [39], which might provide one explanation why seasonal influenza vaccination appeared as an increased risk of pandemic infection [36]. The presence or absence of heterosubtypic immunity does not matter under normal circumstances, but might make big differences in the context of a pandemic caused by reassortment, or zoonotic infection of viruses from another species such as avian H5N1 [39] and H7N9 [40].

There is a strong case to be made for the development of cross-reactive vaccines that induce immunity against different subtypes of influenza, different strains of the same subtypes (broad-spectrum protection) to control seasonal and pandemic influenza [22]. Traditional vaccines target surface proteins haemagglutinin (HA) and neuraminidase (NA), which are specific and always changing so different vaccines have to develop every year. The core of the influenza virus (i.e. the non-glycoproteins) are highly conserved between influenza A virus strains. It is suggested that vaccines that target at highly stable viral gene products such as M2 protein and/or CD8 T cell will elicit cross-reactive antibody and thus heterosubtypic immunity [22], [24], [41], [42], [43]. Development of universal influenza vaccines is one focus of pandemic influenza preparedness, and it is expected that within a decade universal vaccines will become available. The very relevant question is: how effective will these 'universal' vaccines be in controlling the emergence of pandemic influenza via reassortment?

To answer the above questions, in this study we extend our previous population dynamics model of pandemic emergence via reassortment [13] by including seasonal influenza vaccination in the model. We assume two different strains of influenza A virus co-circulate within a human population. Variants introduced by mutation are considered as being identical to their parental strains and thus ignored, with the effect of antigenic drift being reflected in the loss of immunity (cf., [44]). The novel strain is generated only through reassortment from coinfections within a human population, which may mimic what happened in 1957 and 1968 influenza pandemics (e.g., [4], [5], [6]). Because of stochastic behaviour when a novel strain first emerges, we use a stochastic approach to examine the influence of vaccination on the emergence of a pandemic strain via reassortment, and on the number of people infected with the pandemic strain once the pandemic emerges. To test the wide range of immunity response from vaccination and natural infection, differing levels of hetero-subtypic immunity will be considered.

## Model and Methods

### Model assumptions

**Figure 1:**
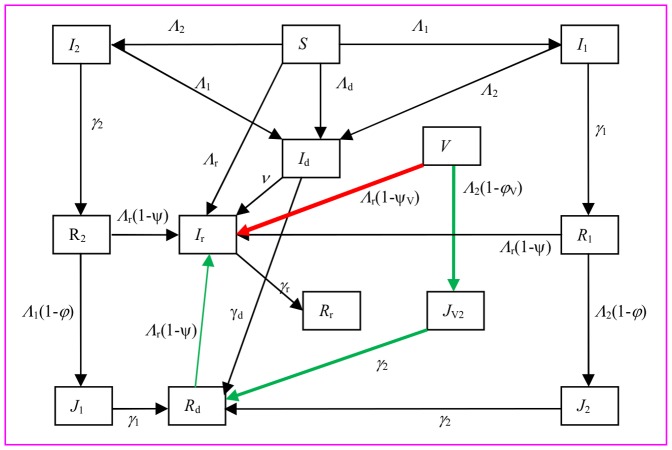
Flow chart of the vaccination model. Arrows indicate transitions and expressions next to arrows show the *per capita* flow rate between compartments. Loss of immunity from recovered or vaccinated to susceptible, births and deaths are not shown. Variables and parameters are explained in Table 1, the force of infection *Λ*_1_, *Λ*_2_, *Λ*_d,_ and *Λ*_r_ are given in equations (2–5).

To study the potential role of vaccination for seasonal influenza in constraining the emergence and spread of novel strains due to reassortment between two co-circulating strains of influenza A virus, we consider a human population which is infected by two strains. Strain 1 is resident, there is a vaccination programme against it, and then strain 2 which is supposed as a minor strain and not included in the vaccine is introduced. Co-circulation of two strains can cause coinfection which then provides chance for reassortment. Empirical studies suggest that two influenza A virus strains of co-infection can simultaneously transmit from person-to-person (e.g., [45]). We assume that strains within dual infection can be transmitted separately or simultaneously. The infectious individuals progress to recover and to become immune to the infecting strain. This immunity wanes over time. Thus we use a Susceptible-Infectious-Recovery-Susceptible (SIRS) model. The human population is classified into 13 compartments to represent infectious processes, and vaccinated, infectious and immune states (see Table 1). Possible processes of transmissions and transitions among 13 compartments are listed in Table 2 and the flow chart of the model is illustrated in Fig. 1.

**Table 1:** Definition of symbols and baseline values of model parameters.

Variable	Description	
*S*	proportion susceptible to all strains	
*V*	proportion vaccinated	
*I_i_*	Proportion singly infected with strain *i* = {1,2}	
*I*_d_	proportion in dual infection with strains 1 and 2	
*I*_r_	proportion in infection with reassortant strain	
*J_i_*	Proportion in secondary infection with strain *i* = {1,2}	
*J*_V2_	Proportion in vaccinated people further infected with strain 2	
*R_i_*	Proportion immune to strain *i* only	
*R*_d_	Proportion immune to both strains 1 and 2	
*R*_r_	Proportion immune to reassortant strain	
parameter	Description	Baseline values
*p*	Proportion of new-borns that were vaccinated	40% [0%,80%]
*β_i_*	Transmission coefficient of strain *i* = {1,2}	0.50 –
*β*_d_	Transmission coefficient of dual infection with both strain 1 and 2	0.10 –
*β*_r_	Transmission coefficient of reassortant strain	0.53 –
*φ_i_*	Factor in transmission coefficient of strain *i* = {1,2} in dual-infection	0.40 [0.0,0.9]
*φ*	Cross-protection conferred by primary infection against endemic strains (reduction in susceptibility)	0.50 [0.0,0.9]
*φ*_V_	Cross-protection conferred by vaccination against endemic strain 2 (reduction in susceptibility)	0.50 [0.0,0.9]
*ψ*	Immunity conferred by previous infection against reassortant strain (reduction in susceptibility)	0.50 [0.0,0.9]
*ψ*_V_	Immunity conferred by vaccination against reassortant strain (reduction in susceptibility)	0.50 [0.0,0.9]
1*/γ_i_*	Infectious period of infection with strain *i* = {1,2}	3.0 day [2.0,4.0]
1*/γ*_d_	Infectious period of dual infection	3.0 day [2.0,4.0]
1*/γ*_r_	Infectious period of infection with reassortant strain	3.0 day [2.0,4.0]
1*/σ*	Duration of natural immunity via primary infection	10.0 years [2.0,20.0]
1*/σ*_V_	Duration of immunization via vaccine	10.0 years [2.0,20.0]
*ν*	Rate of reassortment per coinfection	10^−5^day^−1^ [10^−6^],
1*/µ*	Life span	70.0 years [50.0,80.0]
*N*	Population size	6.3×10^7^ –

Baseline values of model parameters were assumed so the basic reproductive number for both endemic strains are *R*_0_^1^  =  *R*_0_^2^  =  1.50 and for dual infection *R*_0_^d^≈0.3<1, for reassortant strain: *R*_0_^r^  = 1.60>*R*_0_^1^ = *R*_0_^2^. The values given within brackets [] are the range of parameter values considered in sensitivity analyses.

**Table 2:** List of 41 events of the whole model system.

Event	changes	Rate
Birth	*S*←*S*+1	*µ* (1*-p*)*N*
Vaccination	*V*←*V+*1	*µpN*
Death in compartment *S*	*S*←*S*-1	*µS*
Death in compartment *I*_1_	*I*_1_←*I*_1_-1	*µI*_1_
Death in compartment *I*_2_	*I*_2_←*I*_2_-1	*µI*_2_
Death in compartment *I*_d_	*I*_d_←*I*_d_-1	*µI*_d_
Death in compartment *I*_r_	*I*_r_←*I*_r_-1	*µI*_r_
Death in compartment *R*_1_	*R*_1_←*R*_1_-1	*µR*_1_
Death in compartment *R*_2_	*R*_2_←*R*_2_-1	*µR*_2_
Death in compartment *J*_1_	*J*_1_←*J*_1_-1	*µJ*_1_
Death in compartment *J*_2_	*J*_2_←*J*_2_-1	*µJ*_2_
Death in compartment *J*_V2_	*J*_V2_←*J*_V2_-1	*µJ*_V2_
Death in compartment *R*_d_	*R*_d_← *R*_d_ -1	*µR*_d_
Death in compartment *R*_r_	*R*_r_←*R*_r_-1	*µR*_r_
Death in vaccinated V	*V*←*V-1*	*µV*
recovery	*I*_1_←*I*_1_-1, *R*_1_←*R*_1_+1	*γ*_1_ *I*_1_
recovery	*I*_2_←*I*_2_-1, *R*_2_←*R*_2_+1	*γ*_2_ *I*_2_
recovery	*J*_1_←*J*_1_-1, *R*_d_ ←*R*_d_ +1	*γ*_1_ *J*_1_
recovery	*J*_2_←*J*_2_-1, *R*_d_ ←*R*_d_ +1	*γ*_2_ *J*_2_
recovery	*J*_V2_←*J*_V2_-1, *R*_2_←*R*_2_+1	*γ*_2_ *J*_V2_
recovery	*I*_d_←*I*_d_-1, *R*_d_←*R*_d_+1	*γ*_d_ *I*_d_
recovery	*I*_r_←*I*_r_-1, *R*_r_ ←*R*_r_ +1	*γ*_r_ *I*_r_
Loss of immunization	*V*←*V*-1, *S*←*S*+1	*σ*_V_ *V*
Loss of immunity	*R*_1_←*R*_1_-1, *S*←*S*+1	*σR*_1_
Loss of immunity	*R*_2_←*R*_2_-1, *S*←*S*+1	*σR*_2_
Loss of immunity	*R*_d_ ←*R*_d_-1, *S*←*S*+1	*σR*_d_
Loss of immunity	*R*_r_←*R*_r_-1, *S*←*S*+1	*σR*_r_
Reassortment from co-infection	*I*_r_ ←*I*_r_+1, *I*_d_←*I*_d_-1	*νI*_d_
primary infection with strain 1	*I*_1_ ←*I*_1_ *+*1, *S*←*S-*1	*β*_1_ *S*(*I*_1_+*J*_1_+*φ*_1_ *I*_d_)/*N*
primary infection with strain 2	*I*_2_ ←*I*_2_ *+*1, *S*←*S-*1	*β*_2_ *S*(*I*_2_+*J*_2_+*J*_V2_+*φ*_2_ *I*_d_)/*N*
Simultaneous co-infection	*I*_d_ ←*I*_d_ *+*1, *S*←*S-*1	*β*_d_ *SI*_d_/*N*
Primary infection with reassortant strain	*I*_r_←*I*_r_ *+*1, *S*←*S-*1	*β*_r_ *SI*_r_/*N*
Infection in people immune with strain 1 with reassortant strain	*I*_r_←*I*_r_ *+*1, *R*_1_←*R*_1_ *-*1	(1*-ψ*)*β*_r_ *R*_1_ *I*_r_/*N*
Infection in people immune with strain 2 with reassortant strain	*I*_r_←*I*_r_ *+*1, *R*_2_←*R*_2_ *-*1	(1*-ψ*)*β*_r_ *R*_2_ *I*_r_/*N*
Infection in people immune with both strains with reassortant strain	*I*_r_←*I*_r_ *+*1, *R*_d_←*R*_d_ *-*1	(1*-ψ*)*β*_r_ *R*_d_ *I*_r_/*N*
Infection in people vaccinated with reassortant strain	*I*_r_←*I*_r_ *+*1, *V*←*V*-1	(1*-ψ*_V_)*β*_r_ *VI*_r_/*N*
Secondary infection with strain 2 during infectious period	*I*_d_←*I*_d_+1, *I*_1_←*I*_1_ *-*1	*β*_2_ *I*_1_(*I*_2_+*J*_2_+*J*_V2_+*φ*_2_ *I*_d_)/*N*
Secondary infection with strain 1 during infectious period	*I*_d_←*I*_d_+1, *I*_2_←*I*_2_ *-*1	*β*_1_ *I*_2_(*I*_1_+*J*_1_+*φ*_1_ *I*_d_)/*N*
Secondary infection in people immune to strain 2 with strain 1	*J*_1_←*J*_1_ *+*1, *R*_2_←*R*_2_-1	(1-*φ*)*β*_1_ *R*_2_(*I*_1_+*J*_1_+*φ*_1_ *I*_d_)/*N*
Secondary infection in people immune to strain 1 with strain 2	*J*_2_←*J*_2_ *+*1, *R*_1_←*R*_1_-1	(1-*φ*)*β*_2_ *R*_1_(*I*_2_+*J*_2_+*J*_V2_+*φ*_2_ *I*_d_)/*N*
infection in people vaccinated with strain 2	*J*_V2_←*J*_V2_ *+*1, *V*←*V*-1	(1-*φ*_V_)*β*_2_ *V*(*I*_2_+*J*_2_+*J*_V2_+*φ*_2_ *I*_d_)/*N*

Specifically, the following assumptions apply.

1. Neglecting the additional mortality caused by the virulence of infections, births and deaths are assumed to occur at the same rate *µ* so the total population size *N* remains unchanged. Homogeneous mixing of the population is assumed, for simplicity. Hence we ignore age and spatial heterogeneity.
2. During the infectious period, the presence of a strain *i* =  {1, 2} does not affect susceptibility to subsequent infection by the other virus strain 3-*i* [46]. Persons infected with strain 1 but not yet recovered (*I*_1_) can be further infected by strain 2 as easily as those uninfected with strain 1, and vice versa.
3. The interaction between resident strains occurs in two distinct forms: a) *immediate interference*, reducing the transmissibility of strain *i* from dually-infected persons by factor *φ_i_* to *φ_i_β_i_* with 0<*φ_i_*<1, where *β_i_*, *i* =  {1, 2}, is the transmission rate of strain *i* from singly-infected persons to uninfected persons; and b) *post-recovery cross-immunity*, reducing the susceptibility to strain 3-*i* of individuals that have been infected with strain *i* =  {1, 2} but now recovered by factor *φ* with 0<*φ* <1 due to partial cross-immunity.
4. Simultaneous transmission of two strains from dually-infected individuals occurs at a rate *β*_d_ with a constraint *φ*_1_ *β*_1_+*φ*_2_ *β*_2_+*β*_d_≤ min(*β*_1_, *β*_2_), which ensures the low transmissibility of double infection compared to that of single infections.
5. A reassortant strain generated from coinfection is distinct from the two resident strains and their coinfections by having higher transmissibility. However, people who recovered from previous single or/and dual infections with resident strains possess some residual cross-immunity against the reassortant strain and reduce their susceptibility by factor *ψ* with 0<*ψ* <1.
6. A vaccine is used to immunize a proportion of all newborns, conferring full protection against strain 1, but partial protection only against strain 2 and the novel strain from reassortment at rates 1-*φ*_V_ and 1-*ψ*_V_, respectively. Because the vaccine efficacy cannot be 100%, the proportion *p* which is protected is the product of coverage and efficacy (cf., [47]).
7. Since the number of people that are infected with resident strains (i.e., *I*_1_, *I*_2_, *J*_V2_ and *I*_d_) is far smaller than those who are susceptible (*S*) and, generally, those with protective immunity (i.e., *V*, *R*_1_, *R*_2_ and *R*_d_) at any particular time point for influenza diseases, the contacts of individuals infectious with reassortant strain (*I*_r_) with those that are infectious with resident strains are ignored, hence there is no transition from those in *I*_1_, *I*_2_, *J*_V2_ or *I*_d_ to infections with the reassortant strain.

It is worth mentioning that yearly vaccination against seasonal influenza viruses has been recommended for children two years or older in the UK (or 6 months or older in the US), the assumption here is a simple way to model it within a population ignoring age structure (cf., [48]). Further, in reality, people get vaccinated annually (and repeatedly) [38], [49]. The ‘seasonal’ pattern of vaccination has also been simplified because we will only consider the dynamical process within one year period since the second endemic strain was introduced into a human population at endemic with strain 1. Different programmes may cause different proportions under vaccination protection at the time of introduction of the second endemic strain; however, as the results below suggest, it is the strength of cross-immunity that heavily controls the effectiveness of vaccination while the role of vaccination coverage appears relatively weak. Hence we expect that the simple method of modelling vaccination assumed here will be sufficient to approximate the complicated vaccination programme within real populations.

The deterministic version of the model can be specified by a set of differential equations:
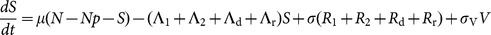


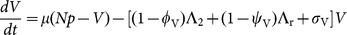


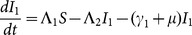


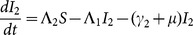


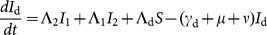


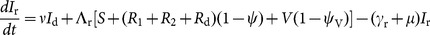
(1)

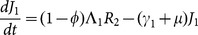


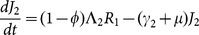


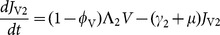


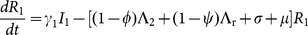


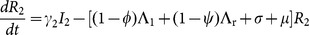


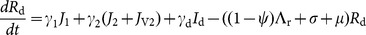


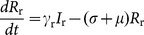



In the above equations, the force of infection of strain *i* =  {1, 2} are
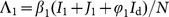
(2)

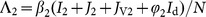
(3)and the force of coinfection and reassortant strain are



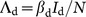
(4)

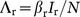
(5)respectively.

### Methods

Surveillance shows that influenza epidemics in any given year are mostly dominated by a single virus A strain [50]. In this study we consider the situation where one influenza virus strain is already endemic and the second strain is introduced. To capture the stochastic features of invasion of the second strain and the generation of reassortant virus strain, we use a Monte Carlo algorithm [51], which tracks the succession of discrete events that change the number of individuals in each compartment. The whole stochastic system is described by 41 possible transition events. Each event occurs at a rate equal to that in the deterministic model (see Table 2). Each compartment is occupied by an integer number of individuals. Denote the sum of all individual event rates by *Ω*. Given initial sizes of compartments, the programme first determines the time of the next event, which follows an exponential distribution with mean 1/*Ω*. The nature of the next event is chosen at random, with each of the 41 events having a probability equal to its own rate divided by *Ω*. For example, the probability *P*_E_ for an event E to occur during the time interval [*t*, *t*+Δ*t*] is



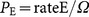
(6)


Here rateE is the rate for event E (see Table 2) at time *t* and Δ*t* ∼ exp(1/*Ω*). After each occurrence, the sizes of the compartments are updated according to the picked events. The simulation programme was coded in Visual c++.

### Empirical information on model parameters

#### Basic features of infection

We first show the basic characteristics of the infectious processes and then explore the different estimates of vaccination efficacy. The basic reproductive number (*R*_0_), defined as the average number of secondary infections that result from the introduction of a single infectious individual into an entirely susceptible population, is an important parameter used to describe the transmissibility of pathogens. *R*_0_ for the influenza A virus is typically in the range 1.2–2.4 while the mean infectious period appears to be shorter than 4 days [52]. For the baseline values of model parameters we assume that among the contacts of dual-infected people with the susceptible, 80% generate single infections equally with strain 1 or strain 2, and 20% to dual infections (so *β*_d_ = 0.20×*β*_1_ = 0.1). By assuming the same infectious period, the baseline values of transmission coefficient are chosen (see Table 1) so that the basic reproductive number of the reassortant strain (*R*_0_^r^)  = 1.6, is slightly higher than that of endemic strains (*R*_0_^1^ = *R*_0_^2^ = 1.5). The assumption of a higher *R*_0_ for the emerging pandemic strain than that of resident strains might be indirectly supported by the observation that the new pandemic strain quickly replaces previously circulating subtypes [18], [19]. The available estimation suggests that on average reassortment occurs at a rate of 10^−5^ per coinfection per day [13].

There are wide ranges for the estimates of cross-immunity from low to very strong [23], [25], [26], [53]. For example, Barry et al. [23] found that the first wave of the 1918-1919 pandemic provided 35–94% protection against clinical illness during the second wave, comparable to that conferred by modern influenza vaccines, which are 50–70% effective against laboratory-confirmed influenza in healthy adults [54], [55]. In view of the estimation that about two thirds of infections have symptoms [56], we assume a level of 50% cross-immunity conferred by primary infection against endemic strains. The duration of immunity has been estimated at 3–20 years [44], [57], [58], [59].

#### Vaccination against endemic strains (φV)

Influenza vaccines are available either as inactivated influenza vaccine (trivalent TIV, and quadrivalent QIV) administered intramuscularly or live attenuated influenza vaccine (LAIV and Q/LAIV) administered intranasally. Vaccinations are targeted at young children and at-risk groups. Many countries are shifting from targeting vaccination at high-risk groups to universal policies with the aim of reducing transmission and thus providing indirect benefits to the population. Influenza vaccines can provide moderate protection against virologically confirmed influenza [55]. A study comparing LAIV and TIV showed that TIV can reduce influenza-related illness by 42%, appearing more effective than LAIV in preventing illness [60]. However, a recent systematic review and meta-analysis from studies published from 1967 to early of 2011 suggests a pooled efficacy of 59% for TIV and 83% for LAIV in reducing the influenza risk of circulating influenza viruses.

Vaccine effectiveness in preventing laboratory-confirmed influenza illness when the vaccine strains are well matched to circulating strains is 70–90% in randomized, placebo-controlled trials conducted among children and young healthy adults; it is lower for the strains that are less matched [61]. For example, Cowling et al. [26] found that TIVs can protect the recipients against seasonal A(H1N1) and A(H3N2); however, Hoskins et al. [62] reported that previous inactivated vaccination may not offer protection against other influenza A strains. That is, annual seasonal flu vaccinations provide weak protection against flu viruses that the vaccine was not designed for [63]. A nested test-negative case control analysis show that the effectiveness of seasonal influenza vaccine in preventing medically attended influenza infection during the 2010/2011 season is about 55% for both type A and type B [64].

#### Vaccination against novel reassortant strains (ΨV)

The systematic reviews and meta-analyses [28] show that the overall cross-protection by TIVs against A(H1N1) pdm09 infection for confirmed illness was 19% (95% confident interval (CI) = 13–42%) with notable heterogeneity. Pebody et al. [65] show that the adjusted vaccine effectiveness was 34% (95% CI: 10–60%) in preventing confirmed A(H1N1) pdm09 infection in the United Kingdom in the 2010/11 season if vaccinated only with monovalent influenza A(H1N1) pdm09 vaccine in the 2009/10 season; 46% (95% CI: 7–69%) if vaccinated only with TIV in the 2010/11 season and 63% (95% CI: 37–78%) if vaccinated in both seasons. Thus this study demonstrates that vaccination with pandemic vaccine in the previous season still provided some residual protection against confirmed A(H1N1) pdm09 infection.

The duration of vaccine-induced immunity appears to be shorter than that induced by natural infection [38]. For example, the duration of immunity induced by TIVs appears to extend beyond one influenza season, lasting between 6 and 12 months [66] while the cross-protective immunity following infection can last more than 5 years [67]. The difference may stem from the different proteins they spur to: vaccinations aim at variable surface proteins while natural infections cause response from conserved internal proteins. Coverage of the seasonal influenza vaccines had reached 30–40% in the general population in US and Canada [68].

## Analyses and Results

In order to investigate the impact of vaccine for seasonal influenza on the emergence probability and the attack rate once it emerges, we assume the following definition for a pandemic emerged from reassortant strains: the total proportion infected with reassortant strain during the one year period since the introduction of the second resident strain (i.e., the 1-year attack rate) must be greater than 5% in view of the observation that the attack rate of seasonal influenza is about 5–10% [39]. We consider a population of the UK size (63 million). The population is assumed to be already endemic with resident strain 1 as
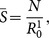


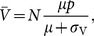
(7)

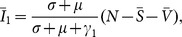


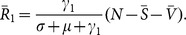



Here *R*_0_^1^ ≡ *β*_1_/(*γ*_1_+*µ*) is the basic reproductive number for strain 1. One single infection seed with strain 2 was then introduced from outside of the population. Ten million stochastic realisations were used to obtain the emergence probability of pandemic via reassortment.

**Figure 2:**
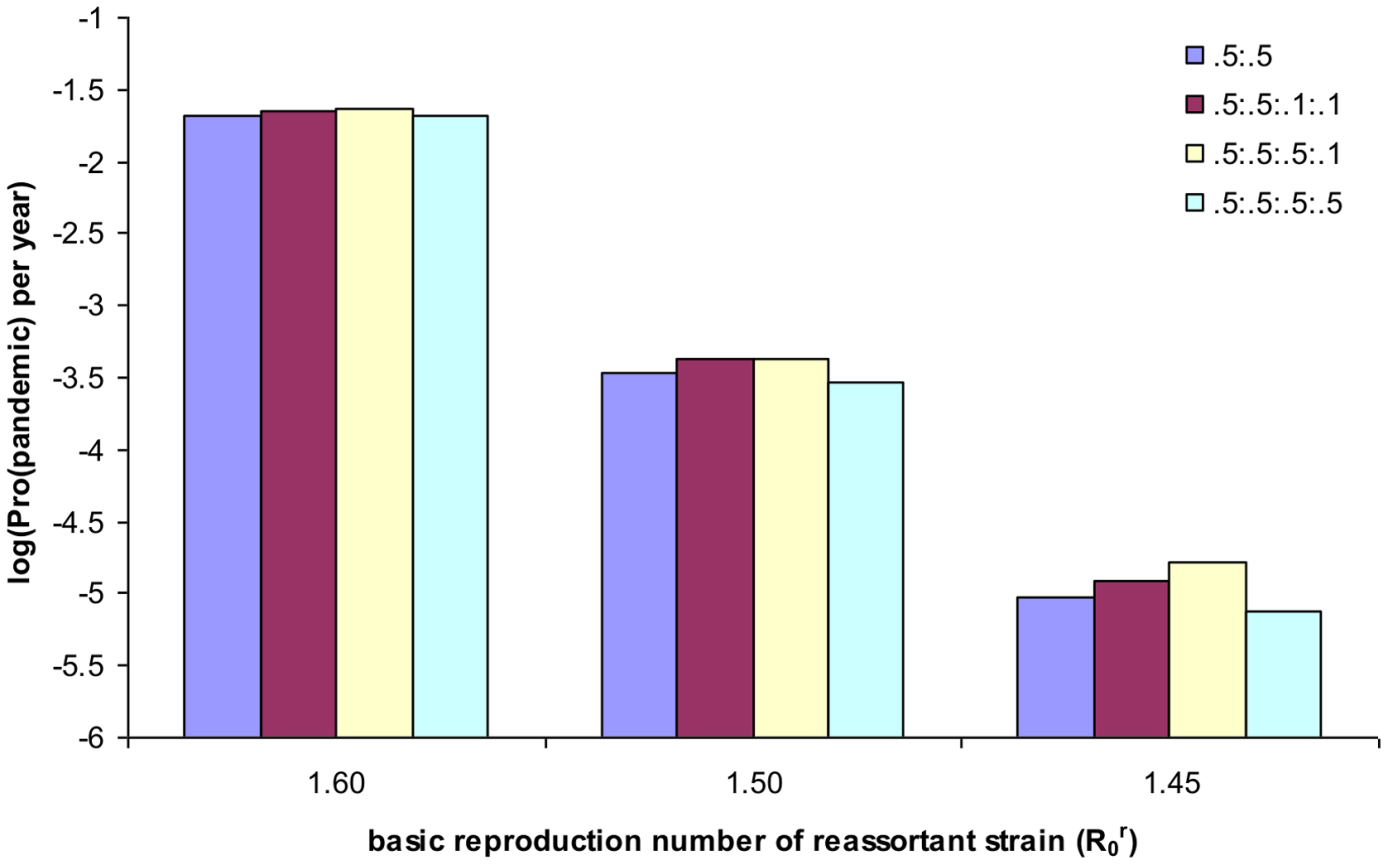
Impact of transmissibility of the oncoming reassortant strain and cross-immunity induced by seasonal flu vaccination on the annual emergence probability of pandemic strain via reassortment. The duration of both infection and vaccination-induced immunity is two years and the values of other model parameters are as in Table 1. Here we consider a situation of cross-immunity structure where the cross-immunity conferred by vaccination are less than or equal to that by natural infection: *φ*:*ψ*:*φ*_V_:*ψ*_V_ *= *0.5:0.5:0.1:0.1 (red), and 0.5:0.5:0.5:0.1 (yellow), 0.5:0.5:0.5:0.5 (green). For comparison, the situations without vaccination (blue) are also shown. As the basic reproduction number (*R*_0_^r^) of reassortant strain reduces, the emergence probability of pandemic decreases rapidly. For example, when *R*_0_^r^ decreases to 1.4, the annual probability of pandemic emergence reduces to below 10^−6^ (data not shown).

Upon the introduction of strain 2, three possible consequences come even under the same values of model parameters: no reassortant strain emerges; reassortant strains are generated but do not persist; reassortant strains emerge and develop into a pandemic (Figure 6 of [13]). Figure 6 of [13] shows that the emerging process is a stuttering scenario due to stochasticity and herd immunity brought up by cross-immunity. Given all other features being the same, the probability of pandemic emergence via reassortment reduces rapidly as the transmissibility of the oncoming reassortant strain decreases and becomes lower than that of the endemic strains (Fig. 2). The emergence probability of pandemic strains depends on two critical processes: the generation of reassortant strains and the increase in the number of infections with reassortant strains. The first process counts on the fraction of coinfection while the other on the effective reproductive number of reassortant strains; both are controlled by the interactions among virus strains. We have discussed how infection-induced cross-immunity against resident strains and novel reassortant strains controls the emergence probability of pandemic influenza via reassortment [13], here we explore how the vaccine-induced cross-immunity influences the emergence probability under different levels of infection-induced immunity.

### Overall effect of vaccination

To illustrate the effect of vaccination, we first consider a simple and ideal situation where all different types of cross-immunity that were generated through vaccination and by primary infection are assumed to be of the same strength. Table 3 shows that the vaccination can help further reduce the emergence probability of pandemic strains. For example, when the cross-immunity against endemic and reassortant strains is 50%, the annual probability of pandemic emergence via reassortment is 0.15% under the situation of no vaccination. When applying vaccine at coverage 40%, the probability reduces to 0.12%, with a relative reduction of 20%. While, at the same vaccination coverage 40%, the probability will be reduced to a vanishingly small value if the cross-immunity increases to 80% (cf., [13]). This indicates that the strength of cross-immunity plays a much stronger role than vaccination coverage does in limiting the probability of pandemic emergence.

**Table 3:** Impact of vaccination on the emergence probability of pandemic strains via reassortment.

Vaccine coverage	Cross-immunity		
	0.2	0.5	0.8
0%	3.21e–2 (6.88e–4)	1.52e–3 (1.98e–4)	5.51e–6 (2.18e–6)
40%	2.77e–2 (1.72e–3)	1.17e–3 (1.46e–4)	2.50e–6 (1.16e–6)
80%	2.24e–2 (1.10e–3)	1.01e–3 (2.26e–4)	1.83e–6 (9.84e–7)

The average probability of pandemic emergence and its standard deviation (in parenthesis) are obtained from ten million realisations of the dynamics processes within one year period since the introduction of the second endemic strain into a population at endemic with strain 1. Here we consider the special situations where vaccination and primary infection induce the same levels of cross-immunity against endemic and reassortant strains (i.e. *φ = ψ  =  φ*_V_ *= ψ*_V_). The values of other model parameters are as in Table 1. The table shows that the levels of cross-immunity heavily control the effectiveness of vaccination while the vaccine coverage plays a much weak role.

**Figure 3:**
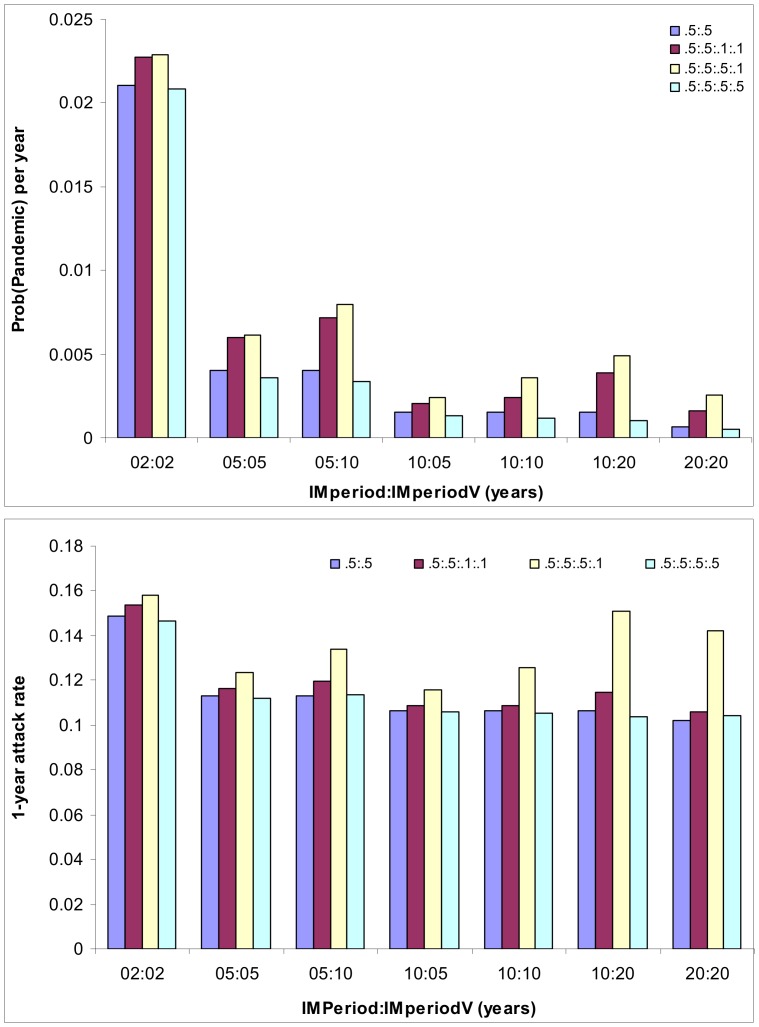
Impact of cross-immunity and its duration induced by seasonal flu vaccination on the annual emergence probability of pandemic strain via reassortment. The values of other model parameters are as in Table 1. The pairs of numbers are the mean durations, in years, of immunity induced by natural infection and vaccination, respectively. Here we consider a situation of cross-immunity structure where the cross-immunity conferred by vaccination are less than or equal to that by natural infection: *φ*:*ψ*:*φ*_V_:*ψ*_V_ *= *0.5:0.5:0.1:0.1 (red), and 0.5:0.5:0.5:0.1 (yellow), 0.5:0.5:0.5:0.5 (green). For comparison, the situations without vaccination (blue) are also shown.

Under the realistic circumstance, cross-immunity generated by vaccination and via infection more likely differs. If the cross-immunity induced by vaccination is weaker, then the probability of pandemic emergence will increase (Figs. 2 and 3). For the example shown in Fig. 3, when the cross-immunity induced by vaccination decreases from *ψ*_V_ *= φ*_V_ = 0.5 to *ψ*_V_ *= φ*_V_ = 0.1, the annual probability of pandemic emergence via reassortment increases from 0.12% to 0.24% for the situation where duration of both types of immunity is 10 years. For the vaccination that induces the cross-immunity of *ψ*_V_ *= *0.1 and *φ*_V_ = 0.5, the emergence probability further increases to 0.36% per year and the 1-year attack rate increases to about 13%. Interestingly, this suggests that when the vaccine-induced cross-immunity against novel reassortant strains (*ψ*_V_) becomes very weak (i.e., 10%), its enhanced level against non-target resident strains actually increases the pandemic risk and attack rate once a pandemic emerges.

Fig. 3 further shows that the duration of immunity can greatly change the probability of pandemic emergence. Consider the example of *ψ*_V_ *= φ*_V_ = 0.5. When the durations of both immunities are 2 years, the probability of pandemic emergence is 2.1% per year; while if the duration of both immunities increases to 10 years, the probability decreases to 0.12% per year. That is, the emergence probability is inversely proportional to the duration of vaccine-derived immunity. Further, Fig. 3 also shows that the effect of duration of vaccine-induced cross-immunity differs from that of infection-induced cross-immunity. Fixing the duration of infection-induced cross-immunity (say at 10 years) and all other parameters remaining the same, for example, the emergence probability of pandemic decreases with the duration of vaccine-induced cross-immunity if vaccine-induced cross-immunity matches or is stronger than that induced by primary infection, but increases with the duration when vaccine-induced cross-immunity is weaker. In contrast, fixing the duration of vaccine-induced cross-immunity (say at 10 years), then the emergence probability of pandemic always decreases with the duration of infection-induced cross-immunity. Their effects on the 1-year attack rate are similar, though comparatively weak (Fig. 3B). These observations are conditional on the implicit assumption that infection-induced immunity is not weaker than that induced by vaccination. This happens because when vaccine-induced cross-immunity is weaker, the longer it lasts, the more people under vaccine-induced immunity protection against the target resident strain (see equation (7)), which increases the effective reproduction number of reassortant strain and the probability for reassortant strain to develop into pandemic.

### Interaction among cross-immunity generated by primary infection and vaccination

**Figure 4:**
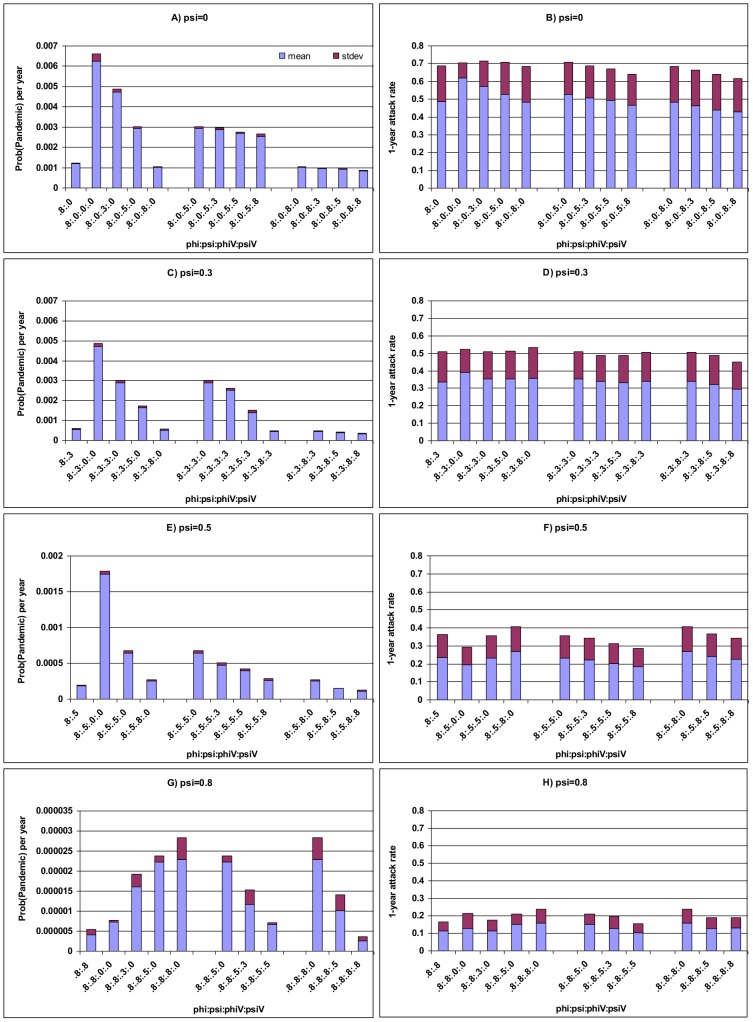
Interaction among cross-immunity generated by primary infection and vaccination: structural cross-immunity on annual emergence probability and size of pandemic. The x-axis *φ:ψ:φ*_V_ *:ψ*_V_ represents the structural cross-immunity. Other parameters as in Table 1. In view of the assumption that vaccine can fully protect against infection with target endemic strain, the situation of *φ*_V_ *= ψ*_V_  = 0 does not imply no effect of vaccination.

Next we scrutinize how the structure of cross-immunity influences the outcome once vaccine was in use. First consider a situation where the primary infection induces much weaker cross-immunity against a reassortant strain than against an endemic strain (i.e. *ψ*<*φ*). To illustrate the possible patterns, we assume a fairly strong cross-immunity against endemic strain of *φ* = 80% (Fig. 4A–4F). As shown in Fig. 3 and Table 3, vaccination can reduce the probability of pandemic emergence once the levels of vaccine-induced cross-immunity match or are stronger than that induced by infection (*ψ*_V_≥*ψ* and *φ*_V_ *= φ*). Further, if *ψ*_V_ = *ψ*, the average size of pandemic remains the same once it begins to emerge. While if *ψ*_V_>*ψ*, then the average size of pandemic will be reduced. This is obvious: given the same level of cross-immunity against endemic strain, stronger cross-immunity against reassortant strain due to vaccination will more reduce the activity of reassortant strain once it emerges; therefore the vaccination decreases both the emergence probability and the size of pandemic.

However, if vaccine-induced cross-immunity is weaker than that induced by infection, vaccination will increase the probability of pandemic emergence. Let us consider an extreme condition where vaccination induces only immunity against the target endemic strain (i.e. *ψ*_V_ *= φ*_V_ = 0), the probability of pandemic emergence will be substantially increased, compared to the situation of no such vaccination (more than 5 times in Fig. 4A, 4C and 4E). In the situations where vaccine can also induce cross-immunity against both resident and reassortant strains (i.e., *φ*_V_ *>*0 and *ψ*_V_>0), the emergence probability will decrease with both *φ*_V_ and *ψ*_V_ (Fig. 4A and 4C); whilst the average size of pandemic always reduces (Fig. 4B and 4D), compared to the above extreme condition. This is easily understood. When people vaccinated have a higher protection against endemic strains, all others being the same, coinfections are rarer, reducing the opportunities for reassortment. If a reassortant strain does emerge, then, its spread is more constrained by a vaccination that incurred a higher level of cross-immunity against reassortant strain and its 1-year attack rate will be reduced.

If the infection-induced cross-immunity against novel reassortant strain is strong but still weaker than that against endemic non-target strain (i.e. *ψ* becomes close to *φ*), some interesting phenomenon comes out. Given a level of vaccine-induced cross-immunity against novel reassortant strains (i.e. fixing *ψ*_V_), increasing the level of vaccine-induced cross-immunity against endemic strains will decrease the emergence probability but increase the 1-year attack rate of pandemic (Fig. 4E and 4F). However, increasing *ψ*_V_ will always decrease both the emergence probability and the 1-year attack rate of the pandemic.

Now we consider a scenario where *ψ* increases to the high level of cross-immunity against resident strains (*φ*) (i.e., 0.8 in Fig. 4G and 4H). As in the above where *ψ* <*φ*, vaccination increases the emergence probability once vaccine-induced cross-immunity is weaker than that induced by infection. Nevertheless, some different behaviours surface. For example, under the extreme vaccination in which *ψ*_V_ *= φ*_V_ = 0, the increase in the emergence probability will be less than 100%, compared to that of no such vaccination (only 78% in Fig. 4G). In the presence of vaccine-induced cross-immunity, all other being the same, *φ*_V_ increases but *ψ*_V_ decreases the emergence probability of pandemic and its 1-year attack rate, compared to the condition where vaccination does not induce any cross-immunity (Fig. 4G and 4H). This is different from the above situation where there is weaker infection-induced cross-immunity against oncoming reassortant strains.

These results suggest that compared to the special situation where vaccination does not induce any cross-immunity, where vaccine-induced cross-immunity against novel reassortant strains occurs (*ψ*_V_) it always helps reduce the pandemic risk; however, the influence of cross-immunity against endemic trains (*φ*_V_) is conditional on the strength of infection-induced cross-immunity against oncoming reassortant strain (*ψ*). When *ψ* is weak, *φ*_V_ decreases both the emergence probability and attack rate of pandemic; if *ψ* becomes strong but still is weaker than *φ*, *φ*_V_ decreases the emergence probability but increases the 1-year attack rate of pandemic. Finally, when *ψ* increases to the level of *φ*, *φ*_V_ increases both the emergence probability and the 1-year attack rate of pandemic. Note that as *ψ* increases from 0.0 in Fig. 4A to 0.8 in Fig. 4G, the emergence probability and size of pandemic decrease; however, their coefficients of variation increase, which suggests the increased stochasticity under the enhanced cross-immunity against reassortant strains.

The above results can be understood as follows. People vaccinated could be infected with reassortant strain through two paths: one via direct contact with people infected by reassortant strain at a rate *Λ*_r_(1-*ψ*_V_) (i.e., the red path in Fig. 1), the other via becoming the recovered *R*_d_ after being infected with endemic strain 2 (i.e., *V*→*J*_V2_→*R*_d_→*I*_r_, at rates *Λ*_2_(1-*φ*_V_) and *Λ*_r_(1-*ψ*) for the 1^st^ and 3^rd^ steps, respectively; the green path). Therefore, given all other conditions remaining unchanged, increasing the vaccine-induced cross-immunity against novel reassortant strains (*ψ*_V_) will decrease the contribution from path 1 and thus reduce the pandemic risk. In contrast, the effect of *φ*_V_, which controls the contribution of path 2, depends on the levels of *ψ*. In the situation where there is much weaker infection-induced cross-immunity against reassortant strain (*ψ*), increasing *φ*_V_ will reduce the contribution through path 2, which decrease the effective reproductive number of reassortant strain and the probability for pandemic emergence. Once emerged, the spread of reassortant strain will be constrained by cross-immunity against both reassortant strains (*ψ*_V_) and resident strains (*φ*_V_), and so will be the size of pandemic (Fig. 4A–4D). For the scenario in which cross-immunity generated from primary infection (*ψ*) becomes strong enough to match or exceed the level of *φ* so that the contribution from path 2 becomes very weak, increasing *φ*_V_ will hardly change the contribution of path 2 but reduce the chance for people vaccinated to get infected with non-target resident strain (i.e. path 2) and allow people vaccinated to stay in class *V* longer. This then increases the contribution of path 1: more people vaccinated to become directly infected with reassortant strain, which hence indirectly increases the overall probability of pandemic emergence (Fig. 4E and 4F). For the situation in between the above (i.e., *ψ* is strong but still weaker than *φ*), there is somewhat weak but yet strong enough contribution from path 2. Increasing *φ*_V_ will directly reduce this bit contribution, which consequently decreases the emergence probability; at the same time it increases the direct contribution from path 1 which results in an increase in 1-year attack rate of pandemic (Fig. 4G and 4H).

## Discussion

Though we cannot manipulate the genetic and antigenic properties of novel reassortant influenza strains, we might be able to change the risk of pandemic emergence in the human population by vaccination. As the pre-requirement for reassortment is co-infection, the risk of pandemic emergence via reassortment would be reduced by reducing the prevalence of infection and particularly the coexistence of multiple strains. Our results show that although vaccination can reduce the chance of coexistence of multiple strains, the actual outcome also depends on the structure of cross-immunity that was naturally generated through primary infection.

If all cross-immunity components generated from vaccination (*φ*_V_ and *ψ*_V_) are at least of the similar strengths to those naturally generated (*φ* and *ψ*), vaccination can reduce the probability of pandemic emergence (Table 3). The probability can be further reduced by prolonging the immunity period (Fig. 3), which implies that repeated vaccination can help reduce coexistence and hence risk of pandemic emergence. To reduce risk of pandemic emergence by controlling the spread of reassortant strains, our investigations indicate that the ideal scheme of vaccination should maintain either strong cross-immunity against both endemic and reassortant strains or at least a strong cross-immunity against reassortant strains (Fig. 4). With seasonal strains prevailing, a large effort has been made to enable the vaccination strong enough to protect against the endemic strains. If vaccination can also build up immunity against reassortant strains [24], [41], the risk of a pandemic will be further reduced. The pandemic can be avoided if the immunization against reassortant strains is strong enough. Therefore if a vaccine that can elicit antibody responses to protect against multiple strains of influenza is available and enough persons are vaccinated, it is possible to effectively control the pandemic at an early stage. Recently, some effort has been made to construct novel approaches for the development of universal influenza vaccines [41], [42]. These might promise to curb seasonal influenza annually and protect people against future pandemics. Pre-pandemic influenza vaccine is an important component of influenza pandemic preparedness plans. Though it cannot constrain the emergence probability of pandemic strain because it becomes available only afterwards [69], its targeted use at the early stage is likely to diminish the attack rate of the novel pandemic influenza.

Owing to the striking diversity in genetic and antigenic properties of reassortant strains, cross-immunity induced by primary infection might not match well to novel reassortant strains. With low levels of infection-induced cross-immunity, the emergence probability of pandemics via reassortment can be high (Table 3; [13]). Our simulation results show that the effectiveness of vaccination in reducing pandemic risk via reassortment is conditional on the infection-induced cross-immunity against reassortant strains (Fig. 4). If considering vaccination policy as a means to reduce pandemic emergence, this reveals the characteristics of the vaccines required and thus raises a challenge for pandemic preparedness via vaccination. In one extreme scenario where there is very weak infection-induced cross-immunity against reassortant strains, universal vaccines that induce strong cross-immunity against common endemic strains and reassortant strains can reduce the probability of pandemic emergence in humans. Further, the attack rate will be reduced with the enhanced level of vaccine-induced cross-immunity against reassortant strains. In the other extreme situation where infection-induced cross-immunity is very strong against reassortant strains, vaccines that induce cross-immunity strongly against common resident strains but weakly against reassortant strains are shown to increase the probability of pandemic emergence (Fig. 4G). The reason for this is apparent: people, who were vaccinated for seasonal influenza and thus highly protected against other common resident strains, remain susceptible to novel reassortant strains, which renders vaccination ineffective against reassortant strains and allows reassortant strains to enjoy a competitive advantage. Moreover, if the reassortant strain emerges, the average attack rate in populations in which such vaccine was applied is higher than the situation where no such vaccine was in use (Fig. 4H). Hence under this scenario, the vaccine for seasonal influenza hardly provides any help in reducing the chance of pandemic emergence and the attack rate if it emerges. For vaccinations that induce weaker cross-immunity than that induced by natural infection, the longer it lasts, the higher the probability that the reassortant strain develops into pandemic is and the larger the attack rate is once a pandemic emerges (Fig. 3). This implies that with such vaccination, the prolonged immunity by repeated vaccination increases the pandemic risk. These analyses suggest that it is the structured cross-immunity generated by both vaccination and primary infection that controls whether vaccination favors or reduces the emergence of pandemic strains though the duration determines the relative probability of pandemic emergence (Fig. 3). Similarly, the vaccination coverage can have a weak effect only on the relative probability of pandemic emergence (Table 3).

A universal vaccine would reduce the risk of pandemic emergence via reassortment. Currently, LAIVs may produce some heterosubtypic immunity but TIVs cannot. A vaccine for seasonal influenza that offers little protection against a reassortant strain might increase the risk of pandemic emergence (cf., [39]). If heterosubtypic immunity induced from vaccination is not broad and strong enough, the effect of vaccination on pandemic emergence depends on the interaction between cross-immunity generated by natural infection and by vaccination. Evaluation of the effectiveness of previous seasonal influenza vaccination in preventing A(H1N1) pdm09 infection has led to a wide range of outcomes: from offering no protection (e.g., [26], [70], [71]), to eliciting partial protection (e.g., [72], [73]), to increasing susceptibility to pandemic influenza [36]. To explain these observations, Mercer et al. [74] proposed a mathematical model incorporating a hypothesised temporary strain-transcending immunity (about 4 months) after infection and concluded that the effect of seasonal vaccination can be explained by the temporary immunity and the timing of the circulation of seasonal and pandemic influenza infection. As they argued, in the Southern hemisphere where pandemic influenza was not preceded by the circulation of seasonal influenza, there was no apparent increased risk from receipt of the seasonal vaccine (e.g., [75]); while in the Northern hemisphere where pandemic influenza circulated soon after seasonal influenza, it was expected to see an apparent increased risk of the seasonal vaccination as observed by Skowronski et al. [36]. However, studies from other Northern hemisphere jurisdictions such as England [76] and other regions of Canada [77] show no effect of seasonal vaccination and hence offer no support to their model.

In this theoretical study, we show that the effect of seasonal vaccination depends on the interaction between cross-immunity induced by vaccination and that acquired through natural infection. As we try to explore the impact of vaccine for seasonal influenza on emergence probability of pandemic via reassortment, we focused on a simple situation: the epidemiological properties of the invader strain are identical to that of the prevailing strain and the oncoming reassortant strain is slightly better transmissible. If the invader strain is different from the prevailing one, the coexistence and coinfection will be reduced, and so does reassortment [13]. If the *R*_0_ of the oncoming reassortant strain is lower than that of the endemic strains, the probability for the reassortant strain to develop into a pandemic will be reduced substantially (Fig. 2) and the nature of stuttering emerging scenario becomes strong under the situation of no vaccination as shown in [13]. This result, on the other hand, hints a higher transmissibility of the pandemic strain from observations of quick spread of the pandemic strain and replacement of prevalent seasonal flu strains. Nevertheless, it is possible that the novel reassortant strain that is of a lower *R*_0_ could be made effectively more transmissible under vaccination for seasonal influenza (Fig. 2). That is, how vaccination for seasonal influenza changes the risk of pandemic influenza under those different situations will still be determined by the interplay between infection-induced and vaccine-induced cross-immunity. The diverse outcomes of seasonal vaccination on pandemic risk from studies on pandemic pdmH1N1 2009 may result from different exposure history and different vaccinations in different populations, suggesting a complicated and variable relationship between immunity induced by natural infection and by vaccination. Combining with this, our results suggest that vaccination for seasonal influenza might effectively protect the human population against endemic influenza virus strains but cannot guarantee its effectiveness in constraining the emergence of pandemic influenza via reassortment.

As the name 'pandemic' suggests, pandemic influenza must take place globally. Though we consider a population of the UK size, the qualitative conclusion obtained should be readily applied to the whole world. Given all other conditions being the same, the overall probability of pandemic emergence via reassortment will increase with the population size. Under the circumstance where all cross-immunity has a duration of 10 years and strength 20%, for example, the probability of pandemic emergence is 0.13 per century for a population size of one million (Table 4 of [13]) and it increases to 3.2 per century for a population of the UK size (63 millions) (Table 3). However, the population size will not change the relationship created by infection-induced and vaccine-induced cross-immunity. Historical data suggest that reassortment may more likely occur in low income regions of the world that are bound to have lower vaccine coverage. This can hardly alter the outcome of our investigation because the role that vaccination coverage plays in limiting the probability of pandemic emergence is quite weak in relation to the strength of vaccine-induced cross-immunity.

Generation and emergence of pandemic strains is a mysterious process, and we do not have decisive evidence about whether reassortment events leading to historical pandemics occurred in humans or other influenza hosts. For simplicity, we only model the scenario where reassortment occurs among humans, which might apply to pandemic strains in 1957 and 1968 (e.g., [4], [5], [6]). To model the pandemic emergence due to a reassortant strain that was generated in other hosts (e.g., pigs) and then jumped to human populations as 2009 pandemic strain [7], dynamic models must include at least two host populations (e.g., [78]). In this study, we ignore age structure and assume homogeneous mixing. In reality, contact patterns among age groups are heterogeneous ([79]) and susceptibility and infectivity vary among ages ([59]). In order to make the conclusions more applicable in practice, these factors should be included, which constitutes a further investigation.
